# CYP1A1 MspI and exon7 gene polymorphisms and lung cancer risk: An updated meta-analysis and review

**DOI:** 10.1186/1756-9966-30-99

**Published:** 2011-10-20

**Authors:** Ping Zhan, Qin Wang, Qian Qian, Shu-Zhen Wei, Li-Ke Yu

**Affiliations:** 1 First Department of Respiratory Medicine, Nanjing Chest Hospital, 215 Guangzhou Road, Nanjing 210029, China; 2 Department of Respiratory Medicine, No. 81 Hospital of PLA, Nanjing, China; 3 Department of Respiratory Medicine, Jinling Hospital, Nanjing University School of Medicine, Nanjing, China

**Keywords:** CYP1A1, Polymorphism, Lung cancer, Susceptibility, Meta-analysis

## Abstract

**Background:**

Many studies have examined the association between the CYP1A1 MspI and exon 7 gene polymorphisms and lung cancer risk in various populations, but their results have been inconsistent.

**Methods:**

To assess this relationship more precisely, a meta-analysis and review were performed. The PubMed, Embase, Web of Science, and CNKI database was searched for case-control studies published up to June 2010. Data were extracted and pooled odds ratios (OR) with 95% confidence intervals (CI) were calculated.

**Results:**

Ultimately, 64 studies, comprising 18,397 subjects from 49 case-control studies of the MspI genotype and 18,518 patients from 40 case-control studies of the exon 7 genotype, were included. A significantly elevated lung cancer risk was associated with 2 MspI genotype variants (for type C vs Type A: OR = 1.26, 95% CI = 1.12-1.42; for types B and C combined vs Type A: OR = 1.20, 95% CI = 1.13-1.28) in overall population. In the stratified analysis, a significant association was found in Asians, Caucasians, lung SCC, lung AC and Male population, not in mixed population, lung SCLC and Female population. However, inconsistent results were observed for CYP1A1 exon7 in our meta-analysis, two variants of the exon 7 polymorphism were associated with a significantly higher risk for lung cancer (for Val/Val vs Ile/Ile: OR = 1.24, 95% CI = 1.09-1.42; for (Ile/Val +Val/Val) vs Ile/Ile: OR = 1.15, 95% CI = 1.07-1.24) in overall population. In the stratified analysis, a significant assocation was found in Asians, Caucasians, lung SCC and Female population, not in mixed population, lung AD, lung SCLC and Male population. Additionally, a significant association was found in smoker population and not found in non-smoker populations for CYP1A1 MspI and exon7 gene.

**Conclusions:**

This meta-analysis suggests that the MspI and exon 7 polymorphisms of CYP1A1 correlate with increased lung cancer susceptibility and there is an interaction between two genotypes of CYP1A1 polymorphism and smoking, but these associations vary in different ethnic populations, histological types of lung caner and gender of case and control population.

## 1. Introduction

Lung cancer remains the most lethal cancer worldwide, despite improvements in diagnostic and therapeutic techniques [1]. Its incidence has not peaked in many parts of world, particularly in China, which has become a major public health challenge all the world [2]. The mechanism of lung carcinogenesis is not understood. Although cigarette smoking is the major cause of lung cancer, not all smokers develop lung cancer [3], which suggests that other causes such as genetic susceptibility might contribute to the variation in individual lung cancer risk [4,5]. Many environmental carcinogens require metabolic activation by drug-metabolizing enzymes. In recent years, several common low-penetrance genes have been implicated as potential lung cancer susceptibility genes.

Cytochrome P450 1A1 (CYP1A1) metabolizes several suspected procarcinogens, particularly polycyclic aromatic hydrocarbons (PAHs), into highly reactive intermediates [6]. These compounds bind to DNA to form adducts, which, if unrepaired, can initiate or accelerate carcinogenesis. Although PAHs are ubiquitous in the environment, notable sources of exposure that cause the greatest concern include smoking, air pollution, diet, and certain occupations [7]. Two functionally important nonsynonymous polymorphisms have been described for the CYP1A1 gene, a base substitution at codon 462 in exon 7, resulting in substitution of isoleucine with valine (Ile462Val (exon 7)) (National Center for Biotechnology Information single nucleotide polymorphism(SNP) identifier rs1048943; adenine (A) to guanine (G) substitution at nucleotide 2455(2455A.G)) and a point mutation (thymine (T) to cytosine (C)) at the MspI site in the 3'-untranslated region (rs4646903;3801T.C) [8]. The MspI restriction site polymorphism resulted in three genotypes: a predominant homozygous m1 allele without the MspI site (genotype A), the heterozygote (genotype B), and a homozygous rare m2 allele with the MspI site (genotype C). The exon 7 restriction site polymorphism resulted in three genotypes: a predominant homozygous (Ile/Ile), the heterozygote (Ile/Val), and the rare homozygous(Val/Val).

An association between CYP1A1 polymorphisms and lung cancer was first reported by Kawajiri and co-workers in 1990 among an Asian study population (Febs Lett 1990;263:131-133)[9], after which many studies analyzed the influence of CYP1A1 polymorphisms on lung cancer risk; no clear consensus, however, was reached. Moreover, 3 meta-analyses have reported conflicting results. Houlston RS [10] found no statistically significant association between the MspI polymorphism and lung cancer risk in 2000, in a meta-analysis performed by Le Marchand L et al. [11] included only 11 studies, the exon 7 polymorphism did not correlate with lung cancer risk. Shi × [12], however, noted a greater risk of lung cancer for CYP1A1 MspI and exon 7 polymorphism carriers in a meta-analysis that included only Chinese population.

A single study might not be powered sufficiently to detect a small effect of the polymorphisms on lung cancer, particularly in relatively small sample sizes. Various types of study populations and study designs might also have contributed to these disparate findings. To clarify the effect of the CYP1A1 polymorphism on the risk for lung cancer, we performed an updated meta-analysis of all eligible case-control studies to date and conducted the subgroup analysis by stratification according to the ethnicity source, histological types of lung caner, gender and smoking status of case and control population.

## 2. Materials and methods

### 2.1 Publication search

We searched for studies in the PubMed, Embase, Web of Science, and CNKI (China National Knowledge Infrastructure) electronic databases to include in this meta-analysis, using the terms "CYP1A1," "Cytochrome P450 1A1," "polymorphism," and "lung cancer." An upper date limit of June, 2010 was applied; no lower date limit was used. The search was performed without any restrictions on language and was focused on studies that had been conducted in humans. We also reviewed the Cochrane Library for relevant articles. Concurrently, the reference lists of reviews and retrieved articles were searched manually. When the same patient population appeared in several publications, only the most recent or complete study was included in this meta-analysis.

### 2.2 Inclusion criteria

For inclusion, the studies must have met the following criteria: they (1) evaluated CYP1A1 gene polymorphisms and lung cancer risk; (2) were case-control studies or nested-case control study; (3) supplied the number of individual genotypes for the CYP1A1 MspI and exon 7 polymorphisms in lung cancer cases and controls, respectively; and (4) demonstrated that the distribution of genotypes among controls were in Hardy-Weinberg equilibrium.

### 2.3 Data extraction

Information was extracted carefully from all eligible publications independently by 2 authors, based on the inclusion criteria above. Disagreements were resolved through a discussion between the 2 authors.

The following data were collected from each study: first author's surname, year of publication, ethnicity, total numbers of cases and controls, and numbers of cases and controls who harbored the MspI and exon 7 genotypes, respectively. If data from any category were not reported in the primary study, the items were designated "not applicable." We did not contact the author of the primary study to request the information. Ethnicities were categorized as Asian, Caucasian, and mixed. Histological type of lung cancer was divided to lung squamous carcinoma (SCC), adenocarcinoma (AC) and small cell lung cancer (SCLC) in our meta-analysis. The definition of smoking history is very complicated. The smoking histories covered different periods if changes in the number of cigarettes smoked per day or type of tobacco products occurred. Cigarette types were classified as filtered or unfiltered commercial products and local traditional hand-made khii yo and yamuan, both unfiltered. According to the general standards, non-smokers were defined as subjects who had smoked less than 100 cigarettes in their lifetime. Although the precise definition of never-smoking status varied slightly among the studies, the smoking status was classified as non-smokers (or never smoker) and smokers (regardless of the extent of smoking) in our meta-analysis. We did not require a minimum number of patients for a study to be included in our meta-analysis.

### 2.4 Statistical analysis

OR (odds ratios) with 95% CIs were used to determine the strength of association between the CYP1A1MspI and exon7 polymorphisms and lung cancer risk. We evaluated this risk with regard to combinations of variants (i.e., type B and type C for MspI and Ile/Val and Val/Val for exon 7) versus the wild-type homozygotes (type A for MspI and Ile/Ile for exon 7).

The pooled ORs for the risk were calculated. Subgroup analyses were performed by ethnicity. Heterogeneity assumptions were assessed by chi-square-based Q-test [13]. A P value greater than 0.10 for the Q-test indicated a lack of heterogeneity among studies, so that the pooled OR estimate of each study was calculated by the fixed-effects model (the Mantel-Haenszel method) [14]. Otherwise, the random-effects model (the DerSimonian and Laird method) was used [15]. In addition, subgroup analysis stratified by ethnicity, gender and histological types of lung caner was also performed.

One-way sensitivity analyses were performed to determine the stability of the results--each individual study in the meta-analysis was omitted to reflect the influence of the individual dataset on the pooled OR [16].

Potential publication biases were estimated by funnel plot, in which the standard error of log (OR) of each study was plotted against its log (OR). An asymmetrical plot suggests a publication bias. Funnel plot asymmetry was assessed by Egger's linear regression test, a linear regression approach that measures the funnel plot asymmetry on a natural logarithm scale of the OR. The significance of the intercept was determined by t-test, as suggested by Egger (P < 0.05 was considered a statistically significant publication bias) [17].

All calculations were performed using STATA, version 10.0 (Stata Corporation, College Station, TX).

## 3. Results

### 3.1 Study characteristics

**Figure 1:**
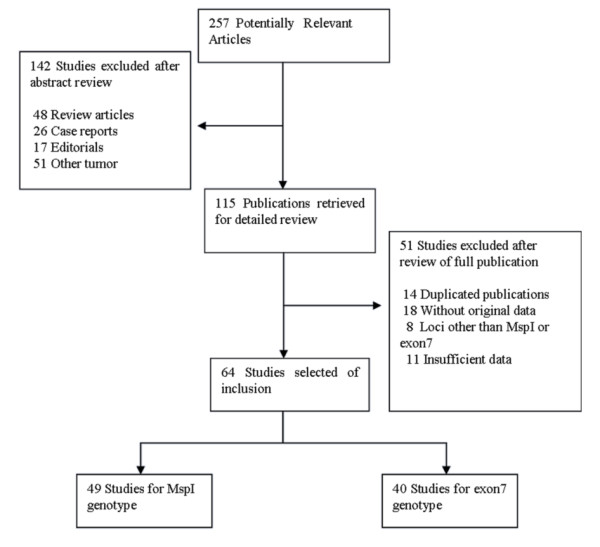
**The flow diagram of search strategy**.

Two hundred and fifty-seven potentially relevant citations were reviewed, and 64 publications met the inclusion criteria and included in our meta-analysis [9,18-80]. Study search process was shown in Figure 1. Table 1 presents the principal characteristics of these studies. For the MspI genotype, 49 studies of 7658 lung cancer cases and 11839 controls were ultimately analyzed. Raimondi's study [58] sorted the data for Caucasians and Asians; therefore, each group in the study was considered separately in the pooled subgroup analyses. For the exon7 polymorphism, 40 studies of 6067 lung cancer cases and 12451 controls were analyzed.

**Table 1:** Distribution of CYP1A1 MspI and exon7 genotypes among lung cancer cases and controls included in this meta-analysis

First author-year	Ethnicity(country of origin)	Total sample size (case/control)	Lung cancer cases of MspI genotype			Controls of MspI genotype			Lung cancer cases of exon7 genotype			Controls of exon7 genotype		
			**Type B**	**Type C**	**Type A**	**Type B**	**Type C**	**Type A**	**Ile/Val**	**Val/Val**	**Ile/Ile**	**Ile/Val**	**Val/Val**	**Ile/Ile**

Kawajiri K-1990	Asia(Japan)	68/104	28	16	24	42	11	51	NA	NA	NA	NA	NA	NA
Tefre T-1991	Caucasian(Norway)	221/212	47	2	172	43	2	167	NA	NA	NA	NA	NA	NA
Hirvonen A-1992	Caucasian(Finnish)	87/121	22	0	65	24	2	95	NA	NA	NA	NA	NA	NA
Shields PG-1993	Mixed populations	56/48	11	2	43	12	3	33	NA	NA	NA	NA	NA	NA
Nakachi K-1993	Asia(Japan)	31/127	7	13	11	55	11	61	11	6	14	44	4	79
Alexandrie AK-1994	Caucasian(Sweden)	296/329	44	4	248	52	1	276	16	0	280	23	0	306
Kelsey K.T -1994	Mixed(African Americans)	72/97	11	1	60	21	2	74	NA	NA	NA	NA	NA	NA
Cantlay AM-1995	Caucasian(Edinburgh)	129/281	NA	NA	NA	NA	NA	NA	21	2	106	33	3	245
Kihara M-1995	Asia(Japan)	97/258	45	16	36	105	41	112	31	5	59	98	14	143
Xu XP-1996	Caucasian(USA)	207/238	35	2	170	48	2	233	NA	NA	NA	NA	NA	NA
Garcia-ClosaM-1997	Mixed populations	416/446	75	4	337	73	4	369	NA	NA	NA	NA	NA	NA
Ishibe N-1997	Mixed(Mexican and African)	171/295	68	12	91	106	35	154	31	7	132	70	20	204
Hong YS-1998	Asia(Korean)	85/63	45	6	34	31	3	29	68	1	16	60	1	2
Taioli E-1998	Mixed populations	105/307	30	9	59	101	18	170	8	1	94	18	0	272
Sugimura H-1998	Asia(Japan)	247/185	NA	NA	NA	NA	NA	NA	94	28	125	84	7	94
Le Marchand L-1998	Mixed populations	341/456	121	35	183	160	44	250	68	6	263	105	13	335
Xue KX-1999	Asia(china)	103/131	NA	NA	NA	NA	NA	NA	31	18	54	36	11	36
Hu YL-1999	Asia(china)	59/132	22	15	22	76	22	34	33	7	19	102	9	21
London SJ-2000	Asia(China)	214/669	NA	NA	NA	NA	NA	NA	39	8	167	130	27	512
Dresler CM-2000	Caucasian(USA)	158/149		37*	121		17*	132	NA	NA	NA	NA	NA	NA
Song N-2001	Asia(China)	217/404	129	28	60	175	56	173	130	9	78	181	13	210
Ratnasinghe D-2001	Caucasian(USA)	282/324	NA	NA	NA	NA	NA	NA	36	3	243	48	3	273
Quinones L-2001	Caucasians(Chile)	60/174	29	10	16	38	16	86	35	10	15	52	14	54
Chen S-2001	Asia(china)	106/106	NA	NA	NA	NA	NA	NA	38	10	58	33	3	70
Xue KX-2001	Asia(china)	106/106	NA	NA	NA	NA	NA	NA	38	10	58	33	3	33
Yin LH-2002	Asia(china)	84/84	34	13	37	38	18	28	NA	NA	NA	NA	NA	NA
Zhou XW-2002	Asia(china)	92/98	43	15	34	34	13	51	66	11	15	65	6	65
Cai XL-2003	Asia(china)	91/138	23	36	32	46	39	53	NA	NA	NA	NA	NA	NA
Kiyohara C-2003	Asia(Japan)	158/259	64	17	77	115	28	116	NA	NA	NA	NA	NA	NA
Taioli E-2003	Mixed populations	109/424 MspI 110/707exon7	20	5	84	75	4	345	16	1	93	70	2	635
Wang J-2003	Asia(china)	162/181	76	22	64	78	38	65	NA	NA	NA	NA	NA	NA
Dialyna IA-2003	Caucasians (Greek)	122/178	28	5	89	45	3	130	NA	NA	NA	NA	NA	NA
Dong CT-2004	Asia(china)	82/91	NA	NA	NA	NA	NA	NA	36	18	28	32	10	32
Gu YF-2004	Asia(china)	180/224		129 *	51		138*	86	NA	NA	NA	NA	NA	NA
Liang GY-2004	Asia(china)	152/152	82	20	50	71	11	70	NA	NA	NA	NA	NA	NA
Chen SD-2004	Asia(china)	58/62	15	23	20	20	18	24	NA	NA	NA	NA	NA	NA
Yang XR-2004	Asia(China)	200/144	NA	NA	NA	NA	NA	NA	96	11	90	39	7	98
Sobti RC-2004	Asia(India)	100/76	45	6	49	29	5	42	67	29	4	53	15	8
Wenzlaff AS-2005	Caucasian(USA)	128/181	35	0	93	30	4	116	5^#^		124	14^#^		134
Wrensch MR-2005	Mixed populations	371/944 MspI 363/930exon7		166*	205		472*	472		64^#^	302		219^#^	711
Ng DP-2005	Asia(Singapore)	126/162	61	22	41	87	19	56	39	13	74	63	7	91
Larsen EJ-2005	Caucasians(Australia)	1050/581	NA	NA	NA	NA	NA	NA	84	8	958	27	2	552
Raimondi S-2005	Caucasians	165/519 MspI 175/723exon7		43*	122		102*	417		32^#^	143		67^#^	656
Raimondi S-2005-2	Asians	46/138 MspI 60/212 exon7		28*	18		95*	43		30^#^	30		96^#^	116
Sreeja L-2005	Asia(Indian)	146/146	53	22	71	45	8	93	NA	NA	NA	NA	NA	NA
Adonis M-2005	Mixed populations	57/103	31	11	15	33	26	44	NA	NA	NA	NA	NA	NA
Belogubova-2006	Caucasians (Russian)	141/450	35	2	104	90	3	357	NA	NA	NA	NA	NA	NA
Li DR-2006	Asia(china)	150/152	NA	NA	NA	NA	NA	NA	104	14	32	105	8	105
Pisani P-2006	Asia(Thailand)	211/408	87	55	26	155	78	53	79	10	78	129	23	135
Yang MH-2007	Asia(Korea)	314/349	NA	NA	NA	NA	NA	NA	116	16	182	111	18	220
Tao WH-2007	Asia(china)	47/94	19	4	24	37	14	43	NA	NA	NA	NA	NA	NA
Cote ML-2007	Mixed populations	354/440	80	5	269	95	6	339	19	0	326	34	6	400
Xia Y-2008	Asia(china)	58/116	36	5	17	58	18	40	NA	NA	NA	NA	NA	NA
Qi XS-2008	Asia(china)	53/72	29	7	17	38	11	23	NA	NA	NA	NA	NA	NA
Yoon KA-2008	Asia(Korea)	213/213	NA	NA	NA	NA	NA	NA	76	10	127	87	10	116
Gallegos-Arreola-2008	Mixed populations	222/248	NA	NA	NA	NA	NA	NA	91	40	91	104	11	133
Shah PP-2008	Asia(India)	200/200		94*	106		63*	137		67^#^	133		44^#^	156
Kumar M-2009	Asia(India)	93/253	NA	NA	NA	NA	NA	NA	17	3	73	40	3	210
Cote ML-2009	Mixed populations	502/523	109	14	373	110	7	402	38	0	464	32	2	489
Honma HN-2009	Mixed populations	200/264	76	11	113	94	9	161	NA	NA	NA	NA	NA	NA
Klinchid J-2009	Asia(Thailand)	85/82		66*	19		66*	16		47^#^	33		42^#^	38
Timofeeva MN-2009	Caucasians (German)	619/1264	NA	NA	NA	NA	NA	NA	248	61	260	545	117	585
Shaffi SM-2009	Asia(India)	109/163		81*	28		85*	78	NA	NA	NA	NA	NA	NA
Jin Y-2010	Asia(China)	124/154		71*	79		70*	80	NA	NA	NA	NA	NA	NA
Wright CM-2010	Caucasians (Australian)	1040/784	219	24	797	128	10	646	103	8	929	40	3	741

NA, not applicable; *, the number of the combined of TypeB and TypeC genetypes;^#^, the number of the combined Ile/Val and Val/Val genotypes.

Of the 64 publications, 50 were published in English and 14 were written in Chinese. The sample sizes ranged from 104 to 1824. All cases were histologically confirmed. The controls were primarily healthy populations and matched for age, ethnicity, and smoking status.

There were 26 groups of Asians, 11 groups of Caucasians, and 12 mixed populations for MspI; for exon7, there were 22 groups of Asians, 10 groups of Caucasians, and 8 mixed populations. All polymorphisms in the control subjects were in Hardy-Weinberg equilibrium.

### 3.2 Meta-analysis results

#### 3.2.1 Association of CYP1A1 MspI variant with lung cancer risk

**Figure 2:**
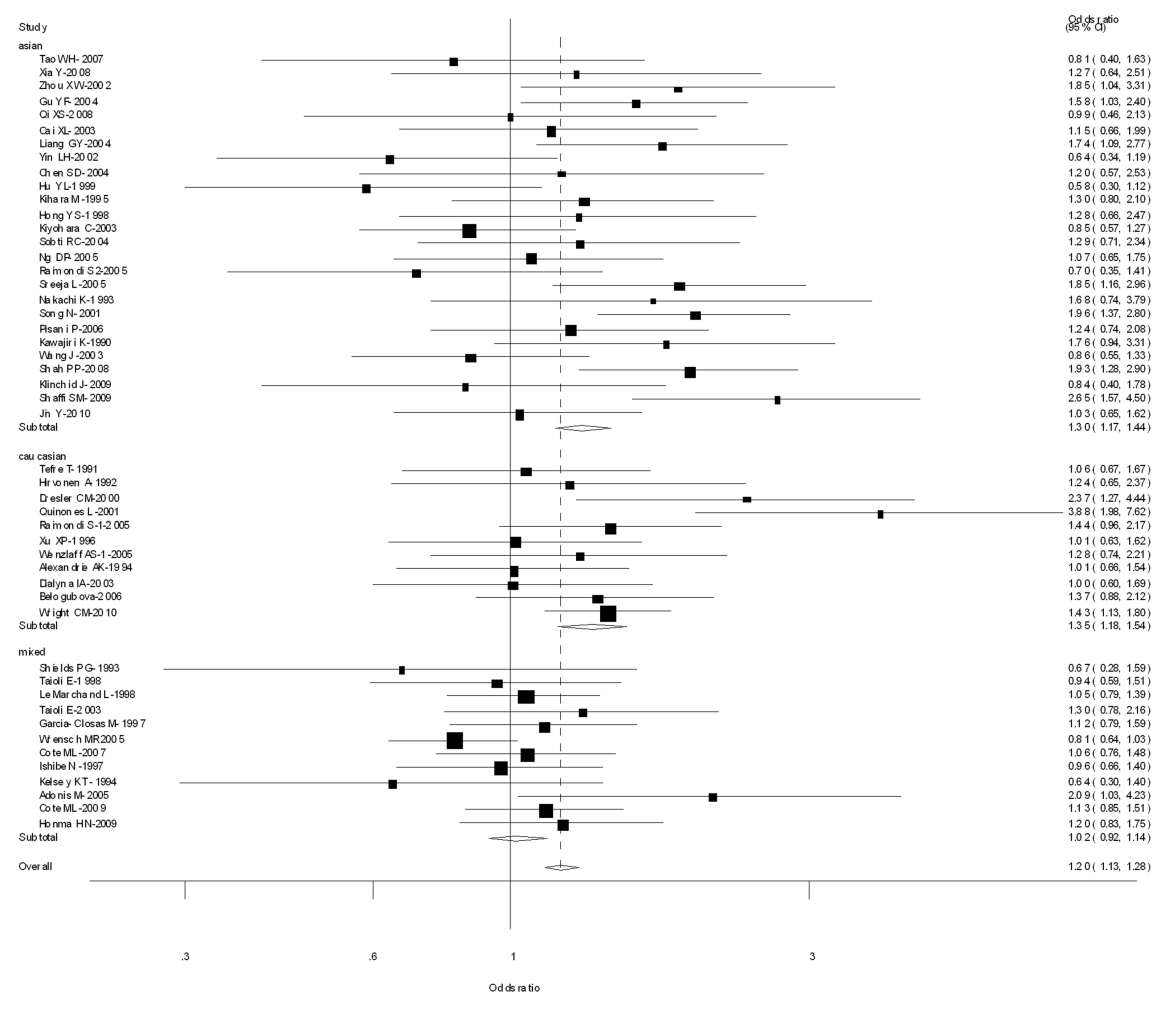
**Forest plot (random-effects model) of lung cancer risk associated with CYP1A1 MspI for the combined types B and C vs Type A**. Each box represents the OR point estimate, and its area is proportional to the weight of the study. The diamond (and broken line) represents the overall summary estimate, with CI represented by its width. The unbroken vertical line is set at the null value (OR = 1.0).

Table 2 lists the primary results. Overall, a significantly elevated risk of lung cancer was associated with 2 variants of CYP1A1 MspI (for Type C vs Type A: OR = 1.26, 95% CI = 1.12-1.42, *P *= 0.003 for heterogeneity; for types B and C combined vs Type A: OR = 1.20, 95% CI = 1.13-1.28, *P *= 0.000 for heterogeneity) (Figure 2).

**Table 2:** Summary ORs for various contrasts of CYP1A1 MspI and exon7 gene polymorphisms in this meta-analysis

Subgroup analysis	MspI genotype			exon7 genotype		
	Contrast	studies	OR(95%) P_h_	Contrast	studies	OR(95%) P_h_
**Total**	Type C vs Type A (TypeB+TypeC) vs Type A	49	1.26(1.12-1.42) 0.003 1.20(1.13-1.28) 0.000	Val/Val vs Ile/Ile (Ile/Val +Val/Val) vs Ile/Ile	40	1.24(1.09-1.42) 0.004 1.15(1.07-1.24) 0.000
Ethnicity						
Asian	Type C vs Type A (TypeB+TypeC) vs Type A	26	1.24(1.12-1.43) 0.004 1.30(1.17-1.44) 0.002	Val/Val vs Ile/Ile (Ile/Val +Val/Val)vs Ile/Ile	22	1.22(1.16-1.59) 0.016 1.21(1.09-1.34) 0.000
Caucasian	Type C vs Type A (TypeB+TypeC) vs Type A	11	1.25(1.09-1.36) 0.053 1.35(1.18-1.54) 0.046	Val/Val vs Ile/Ile (Ile/Val +Val/Val) vs Ile/Ile	10	1.24(1.17-1.43) 0.090 1.28(1.12-1.45) 0.000
Mixed population	Type C vs Type A (TypeB+TypeC) vs Type A	12	1.05(0.89-1.28) 0.140 1.02(0.92-1.14) 0.330	Val/Val vs Ile/Ile (Ile/Val +Val/Val) vs Ile/Ile	8	0.84(0.77-1.03) 0.090 0.92(0.79-1.06) 0.001
**Histological type**						
SCC	Type C vs Type A (TypeB+TypeC) vs Type A	13	1.87(1.58-2.14)0.005 1.93(1.62-2.30) 0.000	Val/Val vs Ile/Ile (Ile/Val +Val/Val) vs Ile/Ile	11	1.38(1.12-1.66) 0.004 1.42(1.18-1.70) 0.007
AC	Type C vs Type A (TypeB+TypeC) vs Type A	12	1.34(1.14-1.56)0.014 1.20(1.01-1.43) 0.000	Val/Val vs Ile/Ile (Ile/Val +Val/Val) vs Ile/Ile	10	0.90(0.72-1.08) 0.005 0.95(0.79-1.15) 0.001
SCLC	Type C vs Type A (TypeB+TypeC) vs Type A	8	0.96(0.70-1.26)0.864 1.06(0.77-1.45) 0.976	Val/Val vs Ile/Ile (Ile/Val +Val/Val) vs Ile/Ile	7	0.84(0.68-1.08)0.068 0.78(0.53-1.14) 0.039
**Gender**						
Male	Type C vs Type A (TypeB+TypeC) vs Type A	3	1.39(1.23-1.79) 0.210 1.46(1.07-1.98) 0.380	Val/Val vs Ile/Ile (Ile/Val +Val/Val) vs Ile/Ile	7	1.18(0.92-1.35) 0.360 1.15(0.96-1.39) 0.298
Female	Type C vs Type A (TypeB+TypeC) vs Type A	7	0.92(0.84-1.16) 0.003 0.85(0.71-1.02) 0.000	Val/Val vs Ile/Ile (Ile/Val +Val/Val) vs Ile/Ile	3	1.29(1.08-1.51) 0.000 1.24(1.05-1.47) 0.002
**Smoking status**		13			10	
Smokers	Type C vs Type A (TypeB+TypeC) vs Type A		1.62(1.33-1.96) 0.000 1.75(1.44-2.13) 0.003	Val/Val vs Ile/Ile (Ile/Val +Val/Val) vs Ile/Ile		1.84(1.36-2.08) 0.003 1.62(1.24-2.11) 0.004
Non-smokers	Type C vs Type A (TypeB+TypeC) vs Type A		1.18(0.96-1.48) 0.086 1.09(0.90-1.33) 0.114	Val/Val vs Ile/Ile (Ile/Val +Val/Val) vs Ile/Ile		1.18(0.96-1.38) 0.080 1.07(0.88-1.31) 0.002

P_h _P value of Q-test for heterogeneity test

In the stratified analysis by ethnicity, significantly increased risks were observed among Asians for both type C vs Type A (OR = 1.24, 95% CI = 1.12-1.43; *P *= 0.004 for heterogeneity), types B and C combined vs Type A (OR = 1.30, 95% CI = 1.17-1.44; *P *= 0.002 for heterogeneity). In Caucasians, there was also significant association in Type C vs Type A (OR = 1.25; 95% CI = 1.09-1.36; *P *= 0.052 for heterogeneity), types B and C combined vs Type A (OR = 1.35; 95% CI = 1.18-1.54; *P *= 0.046 for heterogeneity). However, in mixed populations, no significant associations were observed (Table 2).

**Figure 3:**
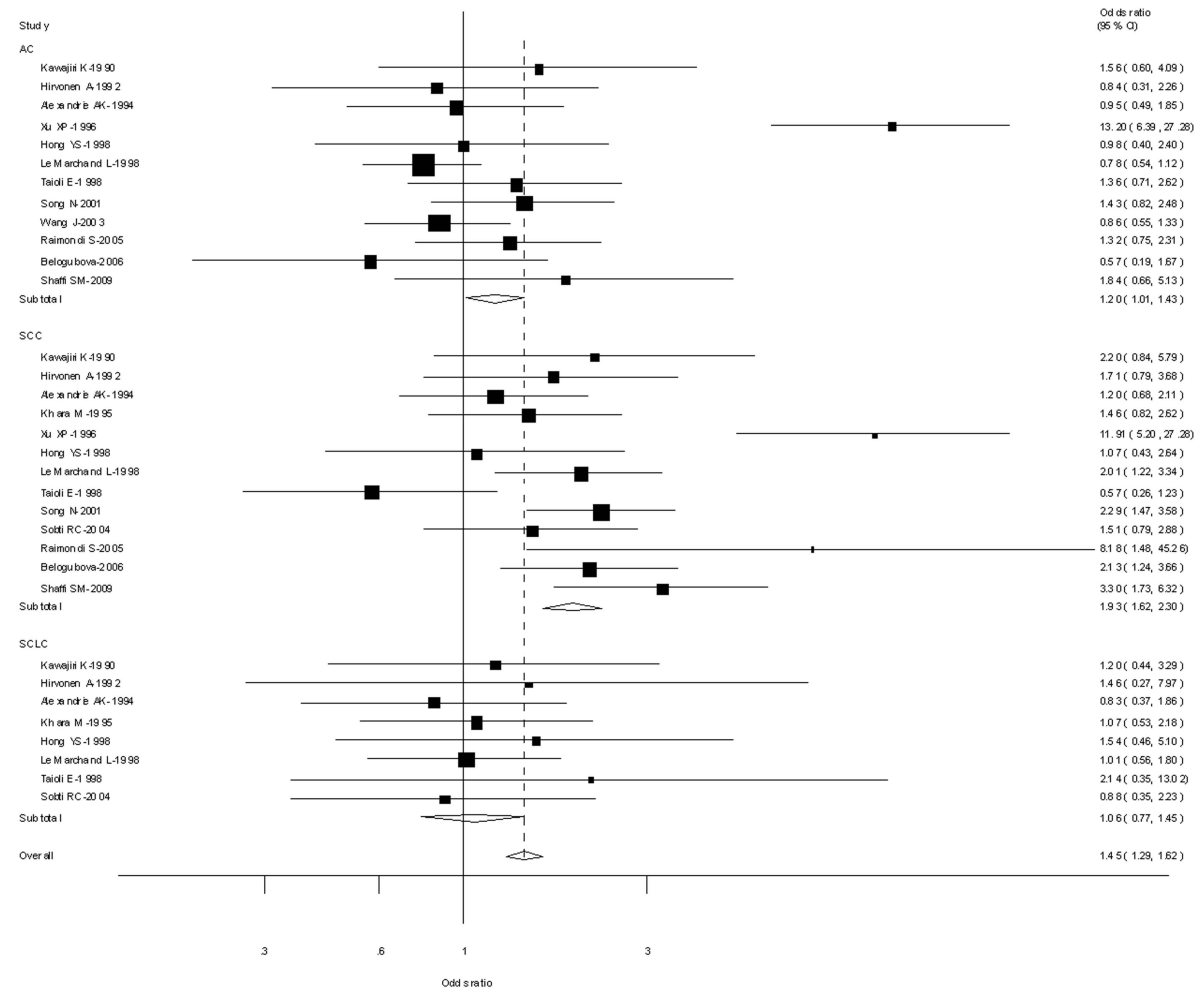
**Forest plot (random-effects model) of lung cancer risk associated with CYP1A1 MspI for the combined types B and C vs Type A stratified by histological types of lung cancer**.

Fourteen [9,19,22,24,26,29,31,32,40,47,53,58,64,78] out of 64 studies examined the association of CYP1A1 MspI genotype and the risk of different histological types of lung cancer including SCC, AC and SCLC. Among lung SCC and lung AC, significantly increased risks were observed for both type C vs Type A, types B and C combined vs Type A. However, among lung SCLC, no significant associations were observed for both type C vs Type A (OR = 0.96; 95% CI = 0.70-1.26; *P *= 0.864 for heterogeneity) or types B and C combined vs Type A (OR = 1.06; 95% CI = 0.77-1.45; *P *= 0.976 for heterogeneity) (Figure 3).

**Figure 4:**
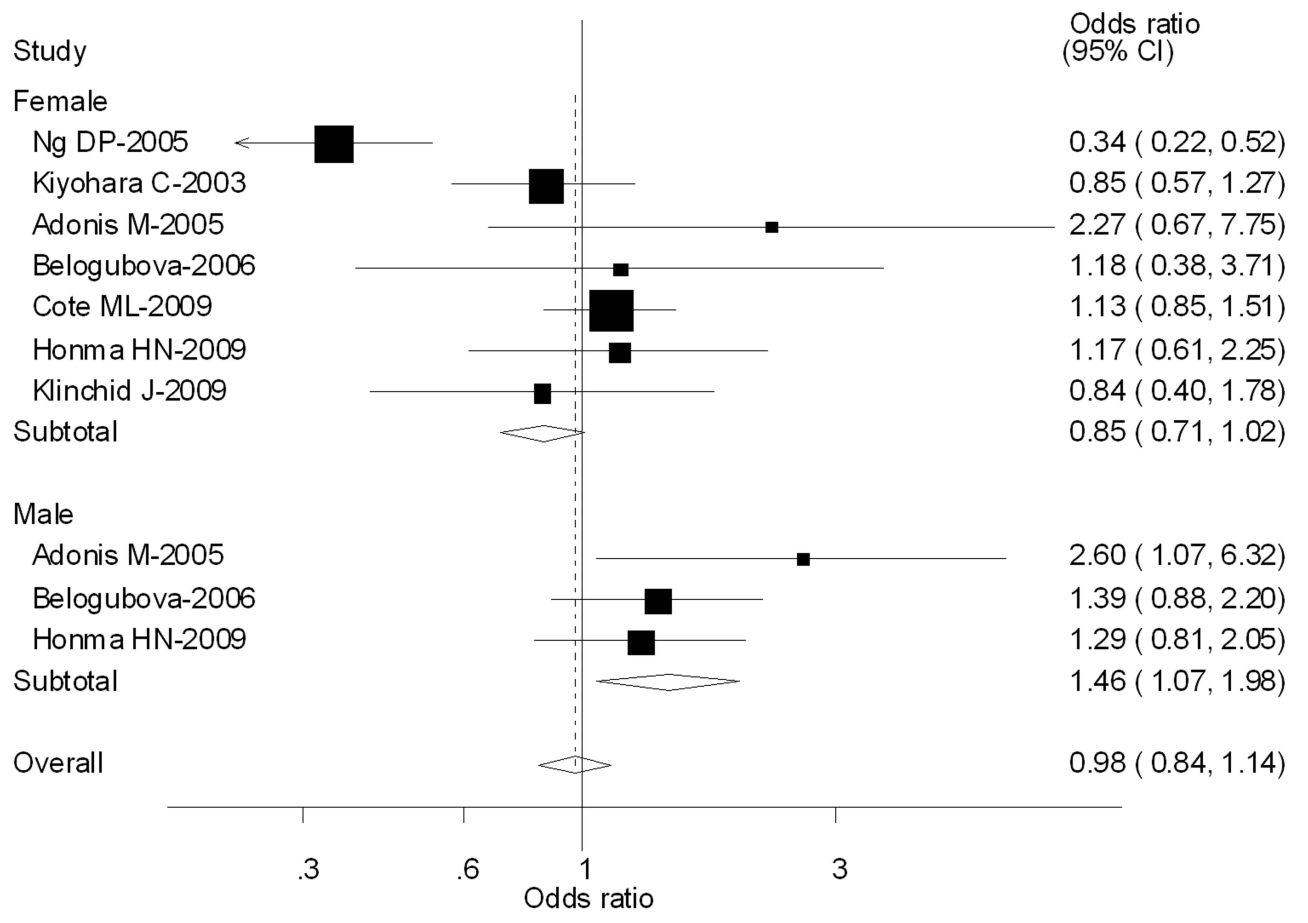
**Forest plot (random-effects model) of lung cancer risk associated with CYP1A1 MspI for the combined types B and C vs Type A stratified by gender of population**.

Seven [45,56,61,64,74-76] out of 64 studies included the association of CYP1A1 MspI genotype and lung caner risk stratified by gender (Male and Female). For Male population (3 studies), significantly increased risks were observed for both type C vs Type A (OR = 1.39; 95% CI = 1.23-1.79; *P *= 0.210 for heterogeneity), types B and C combined vs Type A (OR = 1.46; 95% CI = 1.07-1.98; *P *= 0.380 for heterogeneity). However, for Female population (7 studies), no significant associations were observed for both type C vs Type A (OR = 0.92; 95% CI = 0.84-1.16; *P *= 0.003 for heterogeneity) or types B and C combined vs Type A (OR = 0.85; 95% CI = 0.71-1.02; *P *= 0.000 for heterogeneity) (Figure 4).

**Figure 5:**
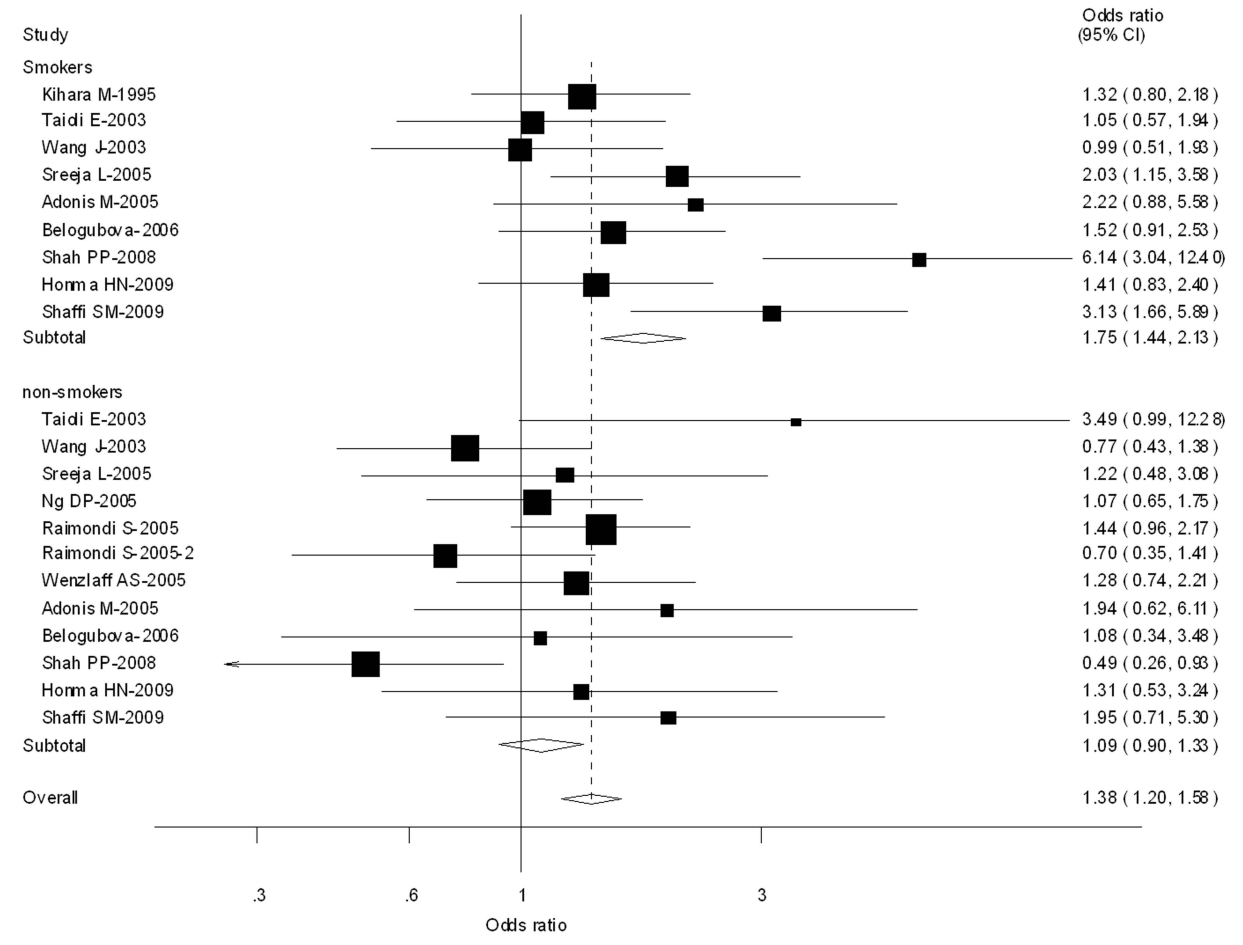
**Forest plot (random-effects model) of lung cancer risk associated with CYP1A1 MspI for the combined types B and C vs Type A stratified by smoking status of population**.

Thirteen [24,31,47,56,59-61,64,72,75,78] out of 64 studies included the association of CYP1A1 MspI genotype and lung caner risk stratified by smoking status (non-smokers or never smokers and smokers). For smokers, significantly increased risks were observed for both type C vs Type A (OR = 1. 62; 95% CI = 1.33-1.96; *P *= 0.000 for heterogeneity), types B and C combined vs Type A (OR = 1.75; 95% CI = 1.44-2.13; *P *= 0.003 for heterogeneity). However, for non-smokers, no significant associations were observed for both type C vs Type A (OR = 1.18; 95% CI = 0.96-1.186; *P *= 0.086 for heterogeneity) or types B and C combined vs Type A (OR = 1.09; 95% CI = 0.90-1.33; *P *= 0.114 for heterogeneity) (Figure 5).

#### 3.2.2 Association of CYP1A1 exon7 variant with lung cancer risk

**Figure 6:**
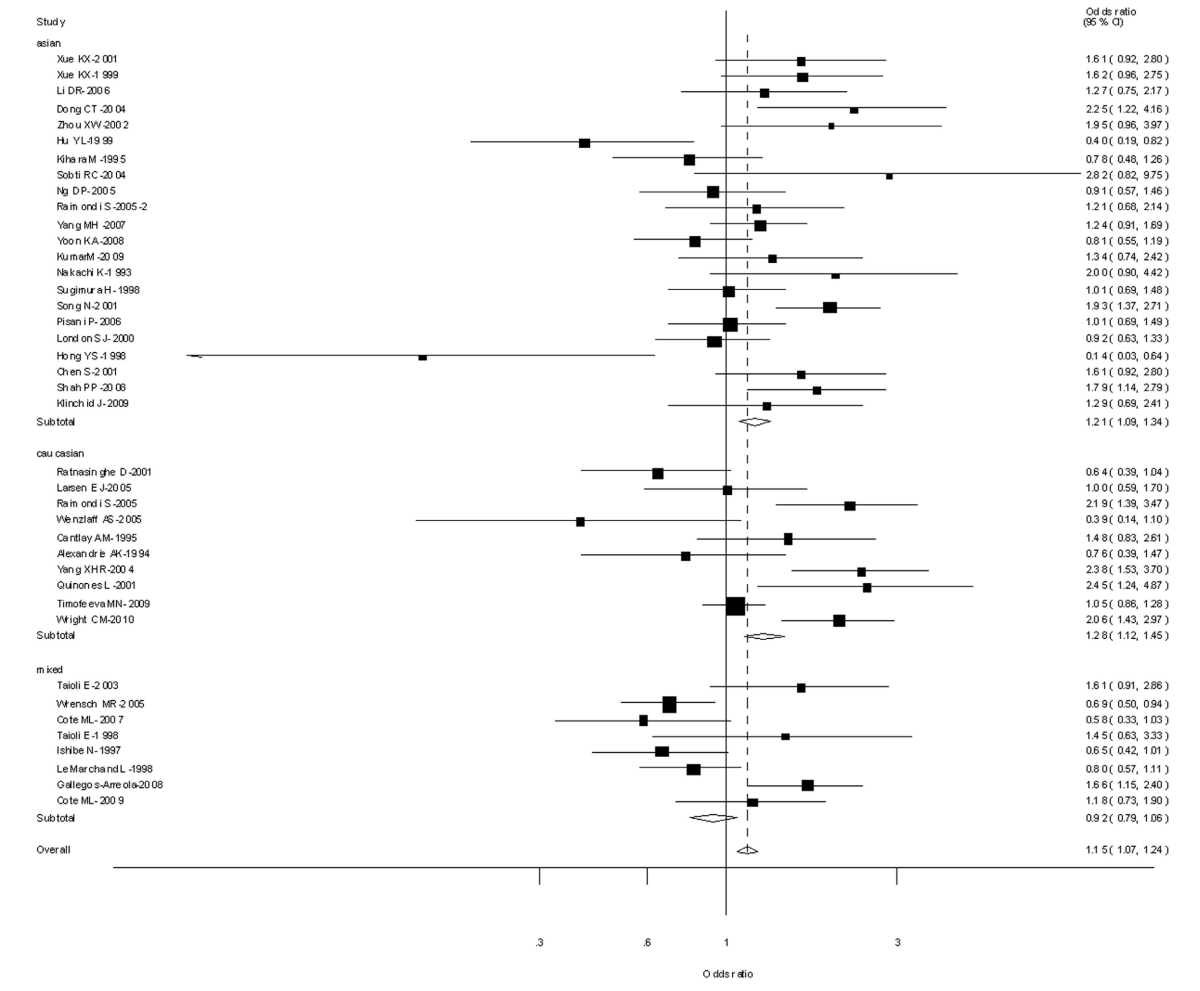
**Forest plot (random-effects model) of lung cancer risk associated with CYP1A1 exon7 genotype for the combined Ile/Val and Val/Val vs Ile/Ile**.

For all studies in the meta-analysis, the genotype, an increased risk for lung cancer was associated with 2 exon7 variants (for Val/Val vs Ile/Ile: OR = 1.24, 95% CI = 1.09-1.42, *P *= 0.004 for heterogeneity; for Ile/Val and Val/Val combined vs Ile/Ile: OR = 1.15, 95% CI = 1.07-1.24, *P *= 0.000 for heterogeneity) (Figure 6).

In the stratified analysis by ethnicity, the risk was higher in Asian carriers of Val/Val vs Ile/Ile (OR = 1.22, 95% CI = 1.16-1.59; *P *= 0.016 for heterogeneity), Ile/Val and Val/Val combined vs Ile/Ile (OR = 1.21, 95% CI = 1.09-1.34; *P *= 0.000 for heterogeneity). A significant association was also observed in Caucasian carriers of Val/Val vs Ile/Ile (OR = 1.24; 95% CI = 1.17-1.43; *P *= 0.090 for heterogeneity) and Ile/Val and Val/Val combined vs Ile/Ile (OR = 1.28; 95% CI = 1.12-1.45; *P *= 0.000 for heterogeneity). However, no significant associations were observed in mixed populations for both Val/Val vs Ile/Ile (OR = 0.84; 95% CI = 0.77-1.03; *P *= 0.090 for heterogeneity) or Ile/Val and Val/Val combined vs Ile/Ile (OR = 0.92; 95% CI = 0.79-1.06; *P *= 0.001 for heterogeneity) (Table 2).

**Figure 7:**
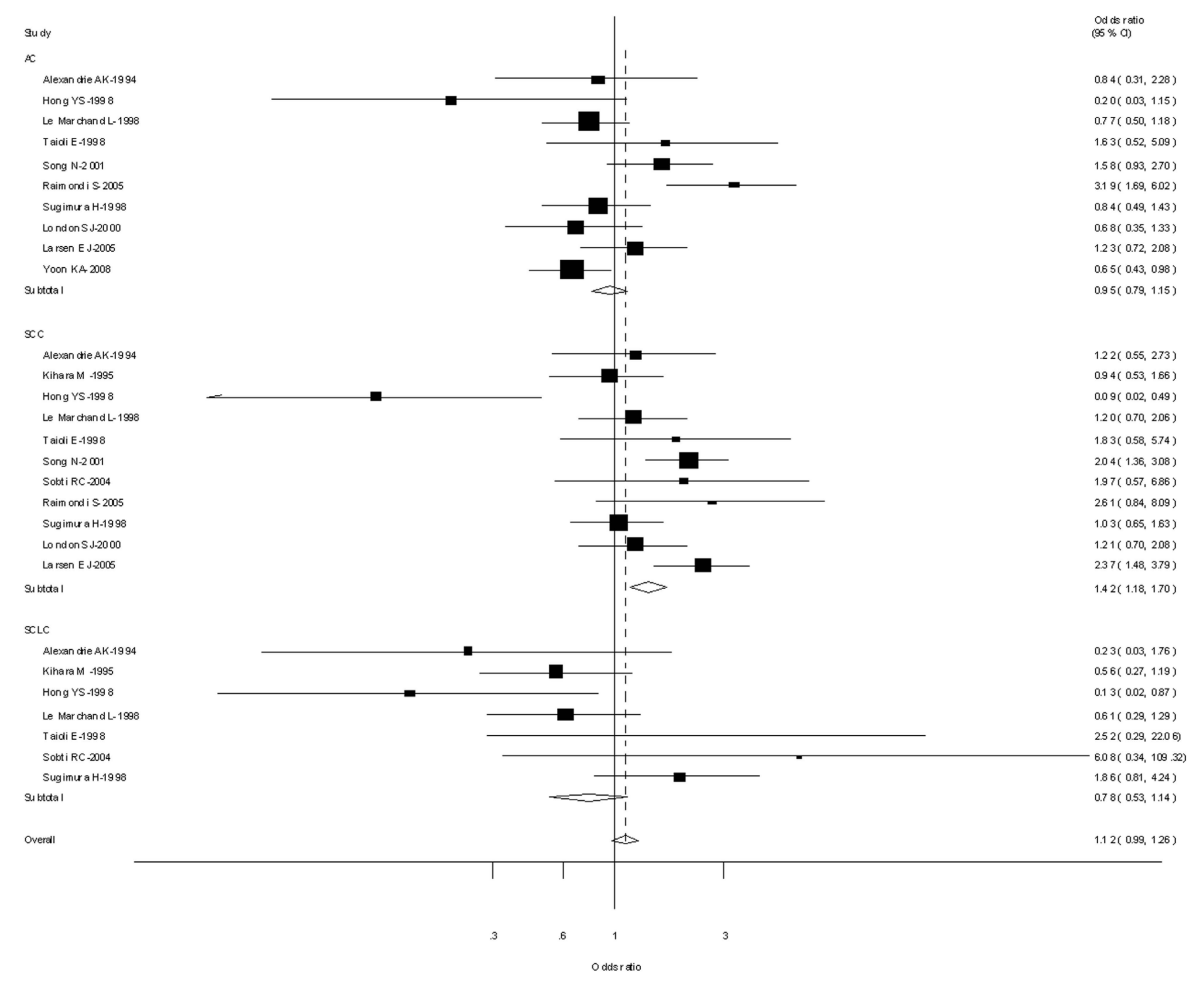
**Forest plot (random-effects model) of lung cancer risk associated with CYP1A1 exon7 genotype for the combined Ile/Val and Val/Val vs Ile/Ile by histological types of lung cancer**.

Twelve [22,24,29-32,36,40,53,57,58,70] out of 64 studies examined the association of CYP1A1 exon 7 genotype and the risk of different histological types of lung cancer including SCC, AC and SCLC. Among lung SCC, significantly increased risks were observed for both Val/Val vs Ile/Ile (OR = 1.38; 95% CI = 1.12-1.66; *P *= 0.004 for heterogeneity) or Ile/Val and Val/Val combined vs Ile/Ile (OR = 1.42; 95% CI = 1.18-1.70; *P *= 0.007 for heterogeneity. However, among lung AC and SCLC, no significant associations were observed for both Val/Val vs Ile/Ile or Ile/Val and Val/Val combined vs Ile/Ile (Figure 7).

**Figure 8:**
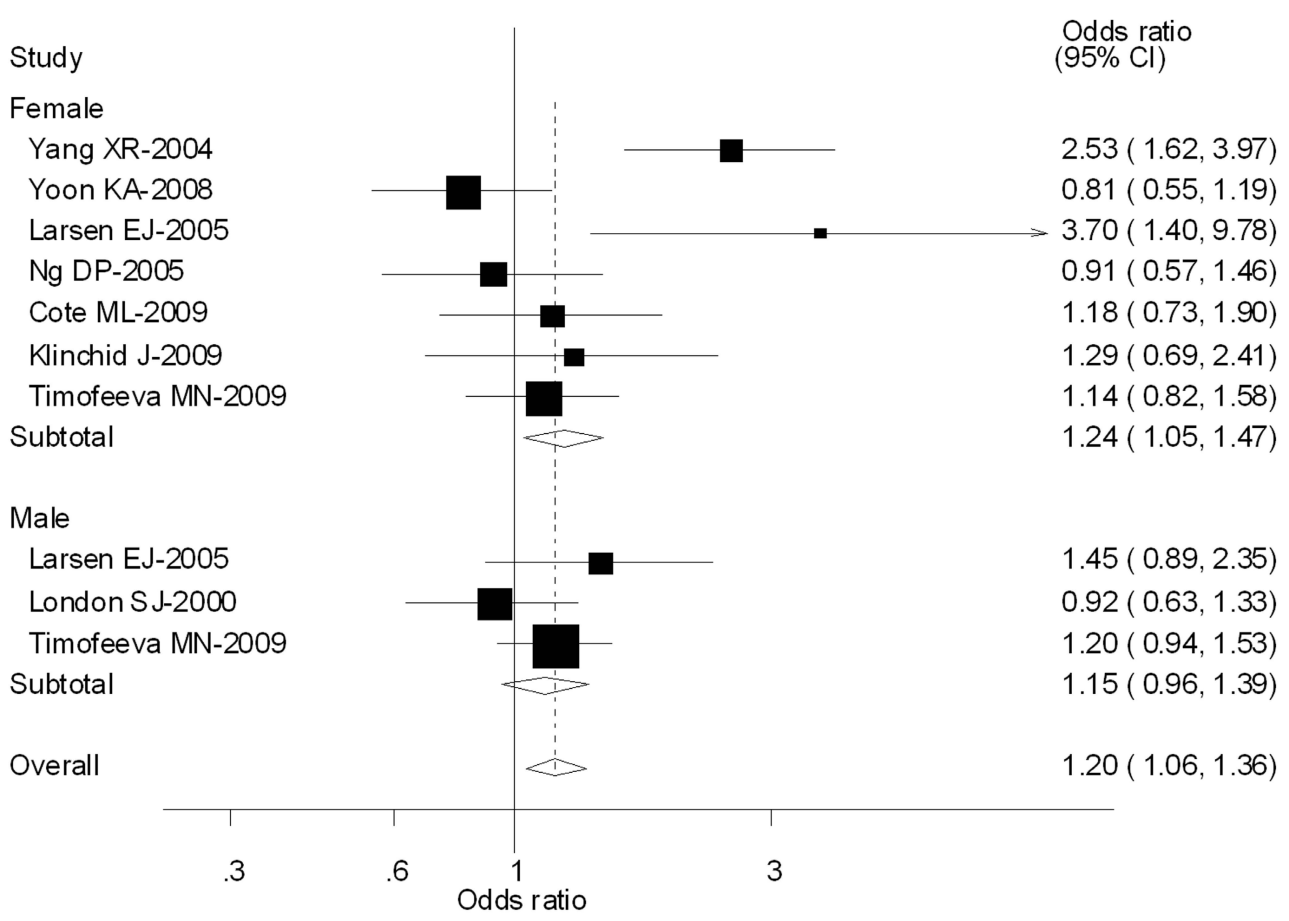
**Forest plot (random-effects model) of lung cancer risk associated with CYP1A1 exon7 genotype for the combined Ile/Val and Val/Val vs Ile/Ile stratified by gender of population**.

Eight [36,54,56,57,70,74,76,77] out of 64 studies included the association of CYP1A1 exon 7 genotype and lung caner risk stratified by gender (Male and Female). For Female population (3 studies), significantly increased risks were observed for both Val/Val vs Ile/Ile (OR = 1.29; 95% CI = 1.08-1.51; *P *= 0.000 for heterogeneity), Ile/Val and Val/Val combined vs Ile/Ile (OR = 1.24; 95% CI = 1.05-1.47; *P *= 0.002 for heterogeneity). However, for Male population (7 studies), no significant associations were observed for both Val/Val vs Ile/Ile (OR = 1.18; 95% CI = 0.92-1.35; *P *= 0.360 for heterogeneity) or Ile/Val and Val/Val combined vs Ile/Ile (OR = 1.15; 95% CI = 0.96-1.39; *P *= 0.298 for heterogeneity) (Figure 8).

**Figure 9:**
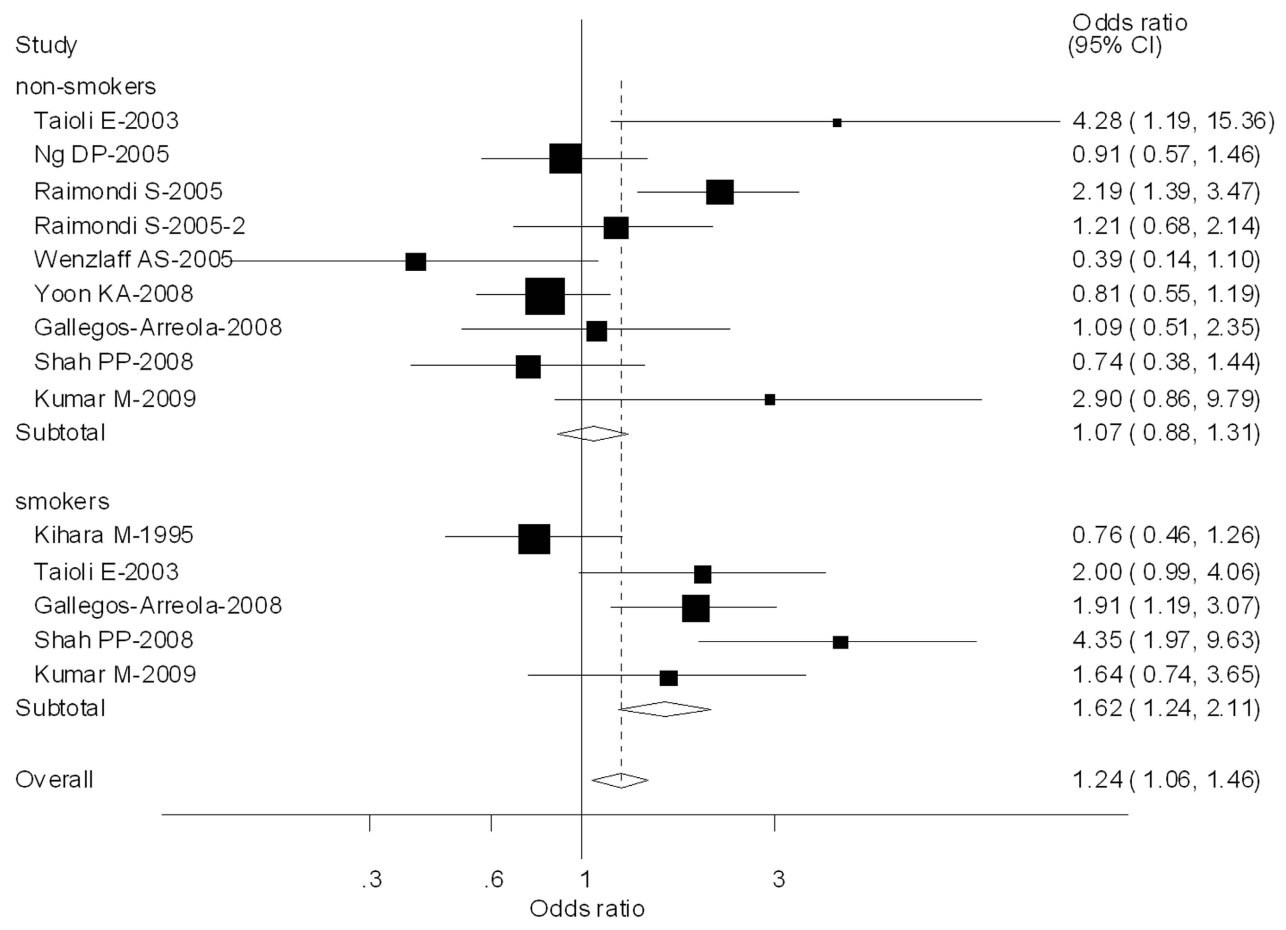
**Forest plot (random-effects model) of lung cancer risk associated with CYP1A1 exon7 genotype for the combined Ile/Val and Val/Val vs Ile/Ile stratified by smoking status of population**.

Ten [24,31,56,60,70-73] out of 64 studies included the association of CYP1A1 exon 7 genotype and lung caner risk stratified by smoking status (non-smokers or never smokers and smokers). For smokers, significantly increased risks were observed for both Val/Val vs Ile/Ile (OR = 1.84; 95% CI = 1.36-2.08; *P *= 0.003 for heterogeneity), Ile/Val and Val/Val combined vs Ile/Ile (OR = 1.62; 95% CI = 1.24-2.11; *P *= 0.004 for heterogeneity). However, for non-smokers, no significant associations were observed for both Val/Val vs Ile/Ile (OR = 1.18; 95% CI = 0.96-1.38; *P *= 0.080 for heterogeneity) or Ile/Val and Val/Val combined vs Ile/Ile (OR = 1.07; 95% CI = 0.88-1.31; *P *= 0.002 for heterogeneity) (Figure 9).

### 3.3 Sensitivity analyses

On omission of each individual study, the corresponding pooled OR was not altered materially (data not shown).

### 3.4 Publication bias

**Figure 10:**
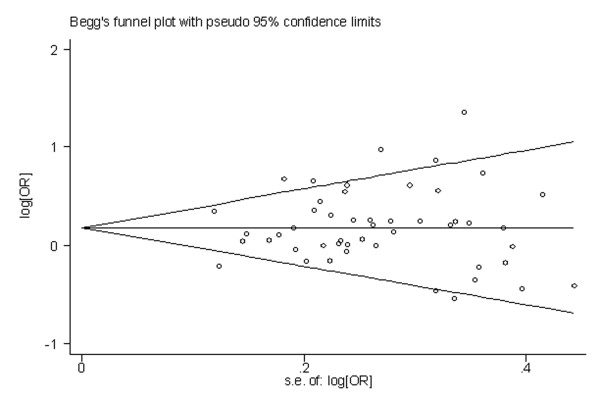
**Begg's funnel plot of CYP1A1 MspI gene polymorphism and lung cancer risk for the combined types B and C vs Type A**.

**Figure 11:**
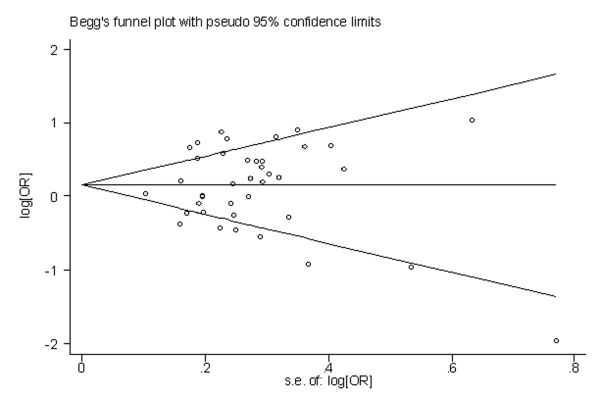
**Begg's funnel plot of CYP1A1exon7 gene polymorphism and lung cancer risk for the combined Ile/Val and Val/Val vs Ile/Ile**.

Begg's funnel plot and Egger's test were performed to identify any publication bias. The funnel plots did not exhibit any patent asymmetry (Figure 10 and 11). By Egger's test--used to provide statistical evidence of funnel plot symmetry--there was no evidence of publication bias (P = 0.558 for publication bias of MspI and P = 0.722 for publication bias of exon 7).

## 4. Discussion

CYP genes are large families of endoplasmic and cytosolic enzymes that catalyze the activation and detoxification, respectively, of reactive electrophilic compounds, including many environmental carcinogens (e.g., benzo[a] pyrene). CYP1A1 is a phase I enzyme that regulates the metabolic activation of major classes of tobacco procarcinogens, such as aromatic amines and PAHs [6]. Thus, it might affect the metabolism of environmental carcinogens and alter the susceptibility to lung cancer. This meta-analysis explored the association between the CYP1A1 MspI and exon7 gene polymorphisms and lung cancer risk, and performed the subgroup analysis stratified by ethnicity, histological types of lung caner, gender and smoking status of case and control population. Our results indicated a significant association between CYP1A1 MspI gene polymorphism and lung cancer risk in Asians, Caucasians, lung SCC, lung AC and Male population, no significant association was found in mixed population, lung SCLC and Female population. Interestingly, inconsistent results were observed for CYP1A1 exon7 polymorphism in our meta-analysis. For the association between CYP1A1 exon7 gene polymorphism and lung cancer risk, a significant assocation was found in Asians, Caucasians, lung SCC and Female population, no significant associations were found in mixed population, lung AD, lung SCLC and Male population. Additionally, a significant association was found in smoker population and not in non-smoker populations for CYP1A1 MspI and exon7 polymorphisms.

When stratified according to ethnicity, a significantly increased risks were identified among Asians and Caucasians for the 2 MspI genotype variants, however no significant association was found in mixed population. For exon 7 polymorphism, the same risk was found in Asians and Caucasians, not in mixed population. These findings indicate that polymorphisms of CYP1A1 MspI and exon 7 polymorphism may be important in specific ethnicity of lung cancer patients. Population stratification is an area of concern, and can lead to spurious evidence for the association between the marker and disease, suggesting a possible role of ethnic differences in genetic backgrounds and the environment they lived in [81]. In fact, the distribution of the less common Val allele of exon 7 genotype varies extensively between different races, with a prevalence of ~25% among East Asians,~5% among Caucasians and ~15% among other population. In addition, in our meta-analysis the between-study heterogeneity was existed in overall population, the subgroup of Asian and Caucasian for MspI and exon 7 genotypes. Therefore, additional studies are warranted to further validate ethnic difference in the effect of this functional polymorphism on lung cancer risk.

There are growing biological and epidemiological data to suggest that different lung cancer pathological subtypes, particularly the two most common, are distinct etiological entities that should be analyzed separately [82]. When subgroup analyses by pathological types were considered, CYPIAl Mspl and exon7 variant alleles were found to be associated with a 1.4-1.9 fold increase in the risk of lung SCC. For lung AC, only CYPIAl Mspl gene polymorphism was significant, however, for lung SCLC, no significant association was found for two genotypes. Our findings were consistent with the Le Marchand L et al study [32] with largest sample sizes of case and control. Le Marchand et al. [32] hypothesized that genetic susceptibility to PAHs predominantly caused lung SCC and nitrosamines caused lung AC. With introduction of filter-tipped cigarettes, probably decreased smokers' exposure to PAHs and increased their exposure to nitrosamines, decreasing trend of SCC, relative to the increase in AC indirectly supports this hypothesis [83]. Different carcinogenic processes may be involved in the genesis of various tumor types because of the presence of functionally different CYP1Al Mspl and exon7 gene polymorphisms. However, the possible molecular mechanisms to explain these histology-specific differences in the risk of lung cancer remain unresolved.

Recent epidemiological and biochemical studies have suggested increased susceptibility to tobacco carcinogens in women compared to men [84-86]. Moreover, CYP1A1 mRNA expression in the lung has been observed to be more than two-fold higher in female smokers compared with male smokers [87]. Another possibly was due to the effect of circulation estrogens, which have been shown to induce expression of PAH-metabolizing enzymes, such as CYP1A1, thereby increasing metabolic activation of carcinogens [88]. In premenopausal women, a higher expression of estrogen can be expected. Estrogen by itself can be involved in carcinogenesis and additionally, it can stimulate expression of CYPs in the female. In our meta-analysis, we found that the effect of CYP1A1 exon7 genotype was observed only in Females, however, for CYP1A1 Mspl the effect was only observed among Males. Our results, along with the previous studies involved above, suggest the difference roles on the two polymorphisms of CYP1A1 genotypes in the susceptibility of lung cancer between Females and Males.

As we know, aside from genetic factor, smoking is the major risk factor of lung cancer. Most studies out of 64 studies reported information on smoking habits of cases and controls, however only sixteen eligible publications provided non-smokers information. Our meta-analysis results showed that a significantly increased risk was found to be associated with the CYP1A1 MspI and exon 7 gene polymorphisms and lung cancer risk in smokers, however, no significant association was found among non-smokers neither CYP1A1 MspI or exon 7 genotype. Tobacco smoke contains many of carcinogens and procarcinogens, such as benzopyrene and nitrosamine. These compounds are metabolized by the phase I enzymes including CYP family enzymes and converted to inactivemetabolites by the phase II enzymes. Our results should indicate the interaction between CYP1A1 MspI and exon 7 gene polymorphisms and smoking in the development of lung carcinoma. However, the association between the extent of smoke exposure and lung caner risk was not clear, further studies with larger sample size are needed to provide insights into the association.

Our data were consistent with the primary results of a previous meta-analysis [89] that showed the MspI and Ile-Val polymorphism of CYP1A1 was a risk factor associated with increased lung cancer susceptibility and these associations varied in different ethnic populations. However, that meta-analysis only conducted the stratified analysis according to ethnicity, smoking and histological types and could not analyze the stratified results in-depth. They could not certify the interaction between smoking status, the major risk fact of lung cancer, and the two genotypes of CYP1A1 polymorphism due to the limitation of included studies. We performed more comprehensive stratified analysis by ethnicity, histological types, smoking status and gender and found the different associations in Male and Female population. We concluded that MspI and exon 7 polymorphisms of CYP1A1 correlated with increased lung cancer susceptibility and there was an interaction between two genotypes of CYP1A1 polymorphism and smoking, but these associations varied in different ethnic populations, histological types and gender of case and control population.

Some limitations of this meta-analysis should be acknowledged. First, heterogeneity can interfere with the interpretation of the results of a meta-analysis. Although we minimized this likelihood by performing a careful search of published studies, using explicit criteria for a study's inclusion and performing strict data extraction and analysis, significant interstudy heterogeneity nevertheless existed in nearly every comparison. The presence of heterogeneity can result from differences in the selection of controls, age distribution, and prevalence of lifestyle factors. Further, only published studies were included in this meta-analysis. The presence of publication bias indicates that non-significant or negative findings might be unpublished. Finally, in the subgroup analyses, different ethnicities were confused with other population, which may bring in some heterogeneity. As studies among the Indians and Africans are currently limited, further studies including a wider spectrum of subjects should be carried to investigate the role of these variants in different populations.

In conclusion, the results of our meta-analysis have provided the comprehensive and convincing evidence that CYP1A1 MspI and exon 7 polymorphisms are an important modifying factor in determining susceptibility to lung cancer. The effect of two genotypes of CYP1A1 polymorphism is diverse by the subgroup analysis stratified by ethnicity, histological types of lung caner and gender of case and control population. More importantly, our study confirms that there is an interaction between two genotypes of CYP1A1 polymorphism and smoking. For future studies, strict selection of patients, well-matched controls and larger sample size will be required. Moreover, gene-gene and gene-environment interactions should also be considered.

## List of abbreviations

CYP1A1: Cytochrome P450 1A1; PAHs: polycyclic aromatic hydrocarbons; CNKI: China National Knowledge Infrastructure; SCC: squamous carcinoma; AC: adenocarcinoma; SCLC: small cell lung cancer; OR: odds ratios; CI: confidence interval

## Competing interests

The authors declare no any conflicts of interest in this work.

## Authors' contributions

PZ and LKY contributed to the conception and design of the study, the analysis and interpretation of data, the revision of the article as well as final approval of the version to be submitted. SZW and QQ participated in the design of the study, performed the statistical analysis, searched and selected the trials, drafted and revised the article. QW participated in the design of the study and helped to revise the article. All authors read and approved the final version of the manuscript.
